# Rheumatoid Vasculitis in Modern Era: A Case Report and Comprehensive Literature Review

**DOI:** 10.7759/cureus.62783

**Published:** 2024-06-20

**Authors:** Anam Ahmad, Farina Tariq, Muhammad Zaheer

**Affiliations:** 1 Internal Medicine, St. Luke's Hospital, Chesterfield, USA; 2 Internal Medicine, Quaid-E-Azam Medical College, Chesterfield, USA; 3 Internal Medicine, St. Louis University School of Medicine, St. Louis, USA

**Keywords:** vasculitis, rheumatoid arthritis, lateral malleolus, non-healing ulcer, rheumatoid vasculitis

## Abstract

Rheumatoid vasculitis (RV) is a rare extraarticular manifestation of severe seropositive rheumatoid arthritis (RA), affecting small and medium vessels and associated with significant morbidity and mortality. The incidence of RV has significantly decreased in the last three decades due to early diagnosis and better management of RA with biologics. Still, the mortality rate remains high and there are insufficient controlled studies guiding RV treatment. Here, we discussed a case of a 75-year-old male who presented with a non-healing ulcer on lateral malleolus without significant joint pain, the workup showed very high titer rheumatoid factor with erosive joint disease raising high clinical suspicion of RV. Skin biopsy was negative for histologic evidence of vasculitis. He had complete healing of the ulcer with prednisone and methotrexate (MTX). This case highlights the importance of promptly recognizing this rare entity and that a negative biopsy does not rule out RV, and appropriate treatment helps decrease morbidity and mortality.

## Introduction

Rheumatoid arthritis (RA) is a chronic systemic inflammatory disease predominantly involving the synovial joints but can have extraarticular manifestations affecting the skin, peripheral nerves, eyes, and heart [1]. Rheumatoid vasculitis (RV) is a rare but severe extraarticular manifestation of RA causing inflammation of small to medium blood vessels with a poor prognosis. RV is usually seen in long-standing, severe, uncontrolled erosive seropositive RA, affecting males more than females [1]. The incidence of RV has markedly decreased due to better control of RA with biologics. Ntatsaki et al. estimated the annual incidence decreased to 3.9 per million (2001-2010) from 9.1 per million (1988-2000) [2]. Here, we present a case of a 75-year-old male with RV manifesting as a non-healing ulcer on the lateral malleolus.

## Case presentation

**Figure 1:**
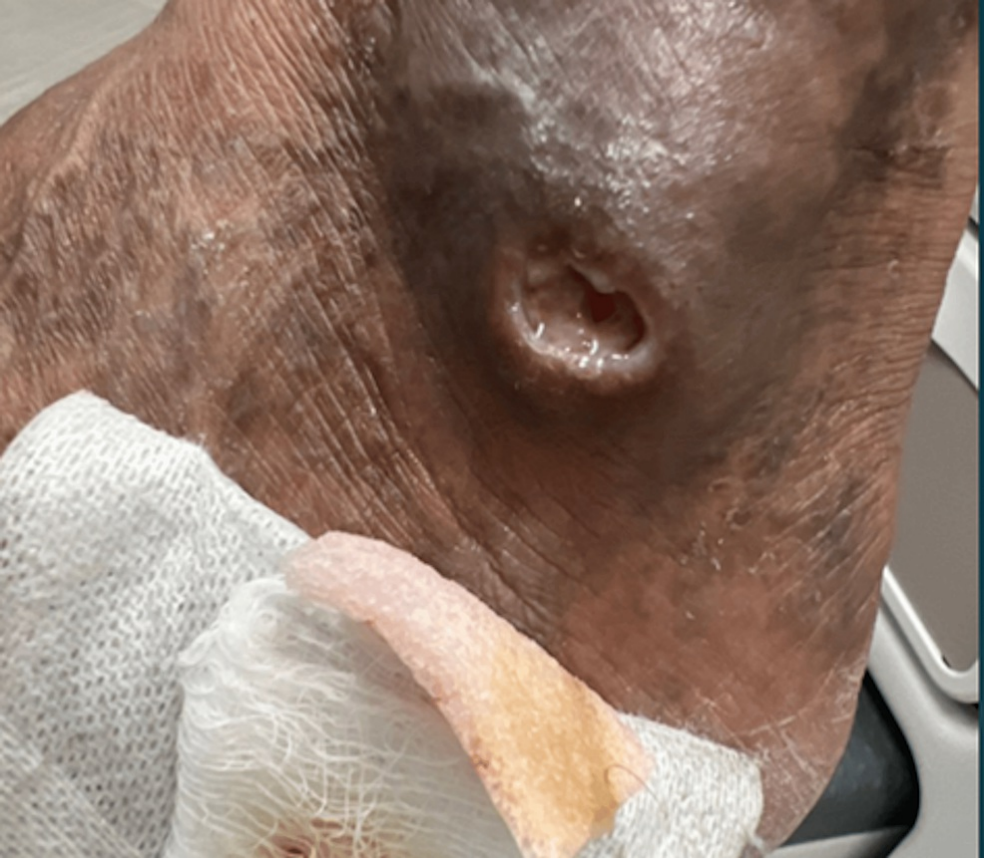
Left lateral malleolus revealing clean-based ulcer.

**Figure 2:**
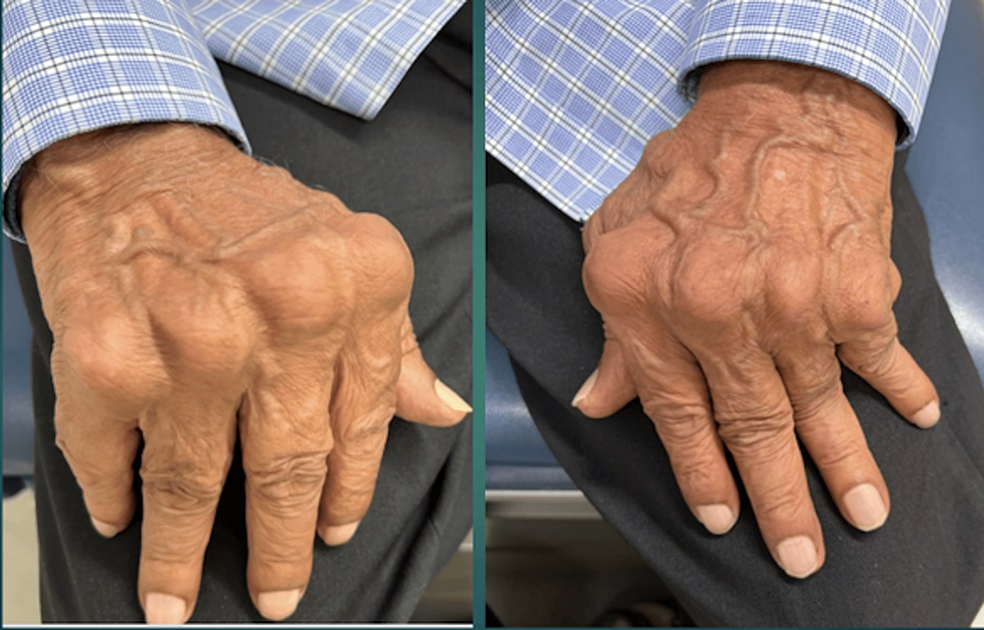
Hands revealing metacarpophalangeal joint subluxation and ulnar deviation of fingers.

A 75-year-old male, active smoker (smoking 0.4 packs a day), with a past medical history significant for hypertension, presented to the rheumatology clinic for evaluation of a three-month history of a non-healing ulcer on the left lateral malleolus and elevated erythrocyte sedimentation rate (ESR) of 107 mm/h. He first developed a papule without any preceding trauma that later got ulcerated. He completed two courses of antibiotics, (doxycycline and trimethoprim/sulfamethoxazole, 14 days each), but the ulcer didn’t heal. The patient denied active joint pain or morning stiffness though he reported a remote history of hand joint pain 10 years ago. A physical examination revealed a clean base ulcer with rolled margins and serous discharge (Figure 1). He had bilateral metacarpophalangeal (MCP) joint subluxation and ulnar deviation (Figure 2). Bilateral posterior tibial and dorsalis pedis pulses were full (2+) on the examination.

**Figure 3:**
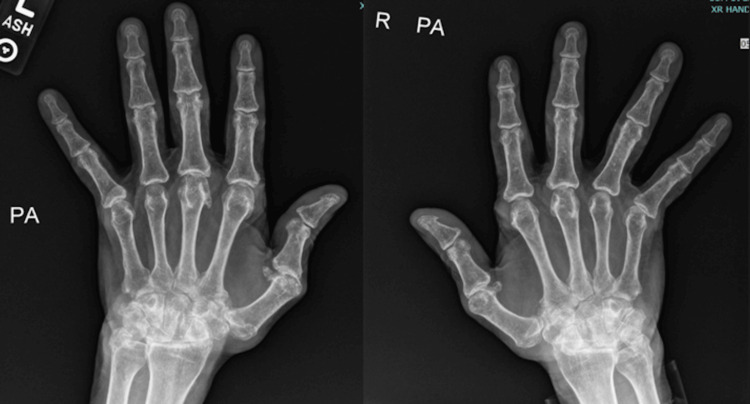
X-ray hands revealing subluxation of the metacarpophalangeal joints.

**Figure 4:**
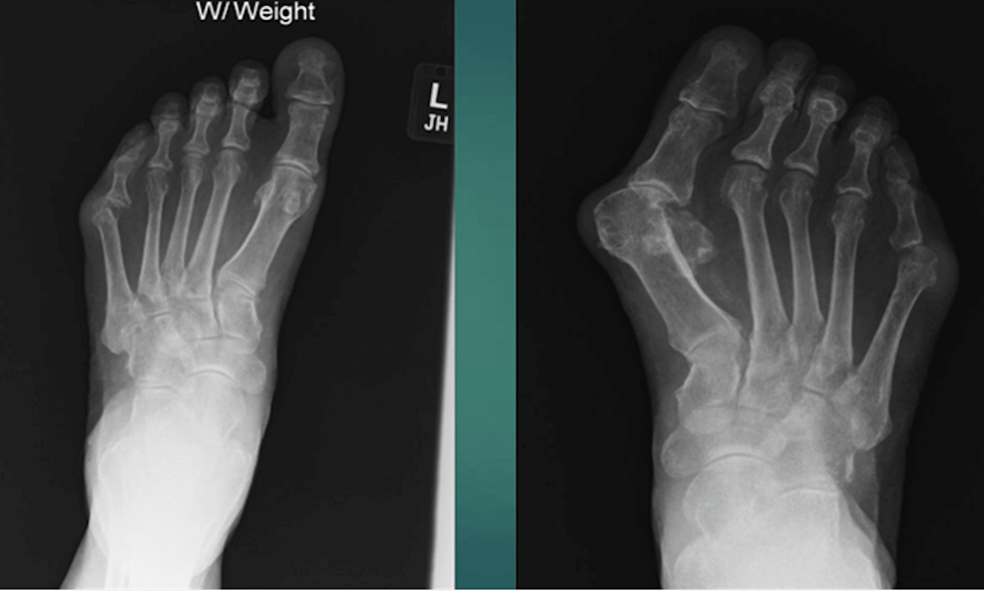
X-rays of feet revealing erosions of bilateral fifth metatarsophalangeal joints.

**Figure 5:**
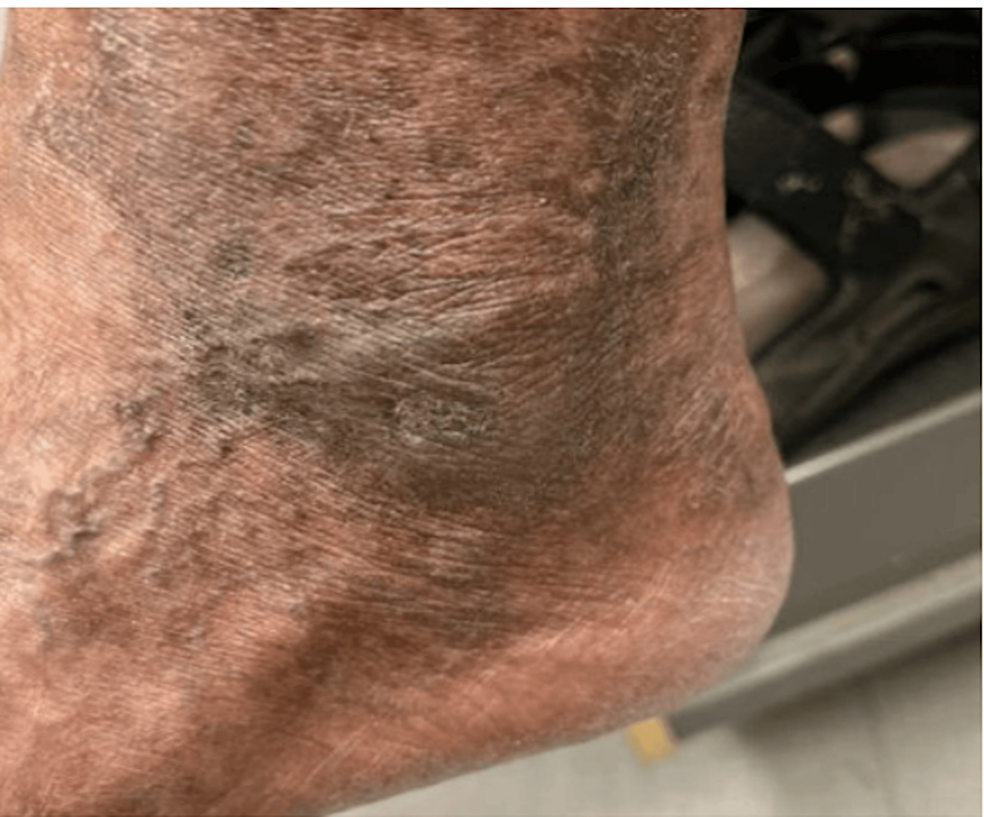
Complete healing of malleolar ulcer after treatment with prednisone and methotrexate.

Laboratory results revealed an elevated erythrocyte sedimentation rate and C-reactive protein with unremarkable complete blood count and complete metabolic panel (Table 1). Considering his hand deformities and the location of the ulcer, RA serologies were checked revealing positive rheumatoid factor and anti-citrullinated protein antibodies (ACPAs). The antinuclear antibody (ANA) comprehensive panel and antineutrophil cytoplasmic antibody (ANCA) titers were negative (Table 1). Ankle brachial index (ABI) was unremarkable. MRI of the left foot was negative for osteomyelitis. The skin biopsy showed no histological features of vasculitis or pyoderma gangrenosum. Tissue Gram stains and cultures were negative. He also had a negative infectious workup for hepatitis B, C, and tuberculosis. Radiographs showed significant arthritis at metacarpophalangeal joints (Figure 3) and erosions in the bilateral fifth metatarsophalangeal joints (Figure 4). Although the biopsy was negative, considering his hand deformities, erosions on X-rays, high titer rheumatoid factor, and location of a non-healing ulcer on the malleolus, a clinical diagnosis of rheumatoid vasculitis was made. He was started on prednisone 1 mg/kg and oral methotrexate (MTX) with complete healing of the ulcer as shown in a couple of months (Figure 5). Prednisone was tapered off over four months and he is currently maintained on methotrexate 20 mg weekly.

**Table 1:** Laboratory investigations of the patient.

Variables	Values	Reference range and units
White blood cells (WBC)	9.3x10^3^/μL	3.5-10.5×10^3^/μL
Absolute neutrophil count (ANC)	3.53×10^3^/μL	1.60-7.50×10^3^/μL
Hemoglobin (Hb)	13.6 g/dL	11.9-15.8 g/dL
Platelets	229x10^3^/μL	150-400×10^3^/μL
Creatinine	0.94 mg/dL	0.56-0.96 mg/dL
Aspartate aminotransferase (AST)	11 U/L	5-34 U/L
Alanine aminotransferase (ALT)	6 U/L	5-55 U/L
Erythrocyte sedimentation rate (ESR)	106 mm/h	0-20 mm/h
C-reactive protein (CRP)	14.9 U/L	<0.5 U/L
Antineutrophilic antibodies (ANA)	Negative	Negative
Extractable nuclear antigen (ENA) profile	Negative	Negative
Rheumatoid factor (RF)	>1800 IU/L	<30 IU/L
Anti-cyclic citrullinated peptide (CCP)	51.9 U/mL	<5 U/mL
Antineutrophilic cytoplasmic antibody (ANCA) panel	Negative	Negative

## Discussion

RV was first described in the 1890s but evolved more in the 1960s when vasculitis with various manifestations was described in patients with RA [3]. RV is typically seen when the inflammatory arthritis is inactive as in our case [4]. Smoking, rheumatoid nodules, and erosive disease are the major risk factors for RV. Infections and medications like tumor necrosis factor (TNF) inhibitors have been proposed as possible triggers [5].

The pathogenesis involves immune complex deposition and complement system activation supported by low complement (C3) levels in RV patients [6]. Overexpression of CD4+CD28 cells has been reported in patients with extraarticular involvement of RA as compared to healthy populations [7] as the overexpression of cellular adhesion molecules (CAM), tumor necrosis factor (TNF) alpha, and other proinflammatory cytokines [8]. All these mechanisms lead to endothelial injury and tissue damage in genetically predisposed patients, homozygous for shared epitope with HLA-DRB1*04 [5].

RV has a heterogeneous presentation with skin involvement being most common (90%), followed by the peripheral nervous system (40%), cardiovascular (30%), eyes (15%), and rarely pulmonary and gastrointestinal systems [1,9]. In skin, RV can manifest as nail fold digital infarcts, palpable purpura, livedo reticularis, non-healing ulcers, or pyoderma gangrenosum. Skin ulcers are deep and normally present on the lower extremities either on the dorsum of the foot or on the malleoli, as opposed to atherosclerotic and venous stasis ulcers [10]. Non-healing ulcers on the malleoli are classic for RV. Fever fatigue, weight loss, and myalgias are subtle symptoms and can herald incipient RV.

The diagnosis of RV is based on clinical presentation, laboratory testing, imaging, and tissue biopsy as indicated. The development of non-healing ulcers and peripheral nervous system involvement like foot drops in RA patients should raise suspicion of RV [10]. Laboratory tests typically show very high titer RF, low C3 levels, and elevated inflammatory markers [1,9]. It is important to rule out vasculitis mimics, especially infections, atherosclerotic disease, malignancy, venous insufficiency, and other systemic vasculitides [11]. Biopsy from accessible sites like skin, sural nerve, muscles, or kidneys in suspected glomerulonephritis can reveal the involvement of all three vessel wall layers with the infiltration of neutrophils and lymphocytes [12]. The biopsy is of high yield and helps make a definite diagnosis, but a negative biopsy doesn’t rule out RV if clinical suspicion is high, as false negative results can occur with insufficient sampling.

Once diagnosed it is important to evaluate the extent of the disease and imaging like computed tomography angiography (CTA) or magnetic resonant angiography (MRA) can be used if there is a clinical suspicion of aortic, mesenteric, or renal involvement [10,13]. Due to the rarity of the disease, there are no randomized controlled trials or evidence-based guidelines for the treatment of RV. Recommendations are based on observational studies and expert opinions. In limited cutaneous involvement like non-healing ulcers, prednisone 0.5-1 mg/kg tapered over 4-8 months is used, and disease-modifying anti-rheumatic drugs (DMARDs) like methotrexate, azathioprine, or mycophenolate mofetil can be added as steroid-sparing agents [14,15]. In case of systemic involvement or organ-threatening diseases like scleritis and mononeuritis multiplex, rituximab or cyclophosphamide should be added to high-dose corticosteroids [16,17]. If the patient is on a TNF inhibitor for RA and develops RV, holding the biologic and monitoring is recommended for possible drug-induced RV [18]. Patient’s age and other comorbid conditions should also be considered for choosing a treatment regimen and smoking cessation should be strongly recommended.

RV is associated with a high mortality rate ranging from 12-14% in one year and 26-60% in five years [2,19]. The involvement of medium vessels and severe systemic disease have been associated with worse outcomes, while the most common cause of death is secondary to infections [20]. The relapse rate is variable and depends on the extent of the disease, organ involvement, and initial treatment duration.

## Conclusions

This case highlights that physicians should be aware that despite a significant decrease in the incidence of RV, in this era of biologics, it is still associated with serious complications and a high mortality rate. Histology is a helpful diagnostic tool, but a negative biopsy cannot rule out the disease. False-negative results in case of the inadequate tissue sample, raise the importance of repeating the biopsy with a deeper section of the tissue. If clinical suspicion is high, prompt recognition and aggressive treatment can help decrease morbidity and mortality.
